# Protective Function of STAT3 in CVB3-Induced Myocarditis

**DOI:** 10.1155/2012/437623

**Published:** 2012-05-24

**Authors:** Diana Lindner, Moritz Hilbrandt, Katharina Marggraf, P. Moritz Becher, Denise Hilfiker-Kleiner, Karin Klingel, Matthias Pauschinger, Heinz-Peter Schultheiss, Carsten Tschöpe, Dirk Westermann

**Affiliations:** ^1^Department of Cardiology and Pneumology, Charité-Universitäts-Medizin Berlin, Campus Benjamin Franklin, 12200 Berlin, Germany; ^2^Department of Cardiology and Angiology, Hannover Medical School, 30625 Hannover, Germany; ^3^Department of Molecular Pathology, University Hospital, 72076 Tübingen, Germany; ^4^Department of Cardiology, Nuremberg Hospital South, 90471 Nürnberg, Germany

## Abstract

The transcription factor signal transducer and activator of transcription 3 (STAT3) is an important mediator of the inflammatory process. We investigated the role of STAT3 in viral myocarditis and its possible role in the development to dilated cardiomyopathy. We used STAT3-deficent mice with a cardiomyocyte-restricted knockout and induced a viral myocarditis using Coxsackievirus B3 (CVB3) which induced a severe inflammation during the acute phase of the viral myocarditis. A complete virus clearance and an attenuated inflammation were examined in both groups WT and STAT3 KO mice 4 weeks after infection, but the cardiac function in STAT3 KO mice was significantly decreased in contrast to the infected WT mice. Interestingly, an increased expression of collagen I was detected in STAT3 KO mice compared to WT mice 4 weeks after CVB3 infection. Furthermore, the matrix degradation was reduced in STAT3 KO mice which might be an explanation for the observed matrix deposition. Consequently, we here demonstrate the protective function of STAT3 in CVB3-induced myocarditis. Since the cardiomyocyte-restricted knockout leads to an increased fibrosis, it can be assumed that STAT3 signalling in cardiomyocytes protects the heart against increased fibrosis through paracrine effects.

## 1. Introduction

Acute viral myocarditis is a frequent cause of sudden cardiac death and can later progress to dilated cardiomyopathy (DCM) due to the chronic inflammatory process. On the one hand, the inflammatory process is needed to control the acute viral infection, but, on the other hand, prolonged inflammation in the subacute phase of the disease will lead to adverse cardiac remodelling. This is mainly characterised with an accumulation of cardiac collagen as well as a deregulation of matrix metalloproteinases, known to be important for collagen degradation and for modulating the inflammatory process [1, 2]. Despite our growing knowledge about viral myocarditis, it remains challenging to diagnose and especially treat patients with viral myocarditis [3, 4]. Therefore, we need to understand more about the inflammatory process in the acute phase of viral myocarditis to tailor future treatment strategies to limit the progression to DCM. 

One of the potent regulators of inflammation is the signal transducer and activator of transcription 3 (STAT3) which is activated in response to extracellular proteins such as cytokines. The members of the IL-6-type cytokine family bind to plasma membrane receptor complexes containing the signal transducing 130 kDa glycoprotein (gp130) that are ubiquitously expressed in most tissues including the heart. Ligand binding to this receptor subsequently leads to the phosphorylation of STAT3 which is then translocated into the nucleus [5]. This family of cytokines is named after the prominent member IL-6 which leads to an increased phosphorylation of STAT3 [6]. Several studies have implicated that STAT3 is essential for hypertrophy and cytoprotection in the heart [7–9]. While its role in acute viral myocarditis is still unknown, it is interesting that the signalling via the gp130/STAT3 pathway is profoundly altered in the myocardium of patients with DCM [10]. It was observed that IL-6 expression as well as STAT3 phosphorylation was decreased in the myocardium of patients with DCM. Interestingly, the myocardial IL-6 expression decreases, whereas the circulating level of IL-6 was increased in patients with heart failure [11, 12]. Moreover, several experimental studies have been performed with a cardiomyocyte-restricted knockout of STAT3 [13]. In general, the cardiomyocyte-restricted STAT3 KO leads to an age-induced fibrosis. Beyond 9 months, the STAT3 KO mice show increased interstitial fibrosis, and, at 12 months, the hearts were dilated [14, 15], suggesting a role for STAT3 in cardiac remodelling and the progression to DCM.

Here, we study the effect of cardiomyocyte-restricted knockout of STAT3 in viral myocarditis to evaluate its role during inflammation as well as adverse cardiac remodelling in experimental viral myocarditis.

## 2. Material and Methods

### 2.1. Study Design

Mice with the cardiomyocyte-restricted STAT3 deletion were generated on a CB6FI genetic background as described previously [14] and kept under standard conditions. Male STAT3 KO and WT animals were infected with 10^6^ plaque-forming units of CVB3 intraperitoneally (all mice were 6 weeks old at the day of infection). Infected mice were compared with saline-treated mice of both groups 10 and 28 days after infection. This investigation conforms to the Guide for the Care and Use of Laboratory Animals published by the US NIH (NIH Publication number 85-23, revised 1996).

### 2.2. Hemodynamic Measurements and Surgical Procedures

Four weeks after infection with CVB3, all animals were anesthetized (thiopental 125 mg/g i.p.), intubated, and artificially ventilated. A 1.2 F-mircoconductance pressure catheter (SciSence, Ontario, Canada) was positioned in the left ventricle via the right carotid artery for continuous registration of pressure-volume loops in a closed-chest model as described previously [16].

Global function was quantified by heart rate (bpm), cardiac output (mL/min), stroke volume (*μ*L), stroke work (*μ*L · mmHg), and ejection fraction (%). Systolic function was assessed by end systolic pressure, P_es_ (mmHg), left ventricular contractility dP/dt_max⁡_ (mmHg/s), and end systolic volume V_es_ (*μ*L). Diastolic performance was measured by end diastolic pressure P_ed_ (mmHg), left ventricular relaxation dP/dt_min⁡_ (mmHg/s), left ventricular relaxation time Tau (ms), and end diastolic volume V_ed_ (*μ*L). 

Hearts of sacrificed animals were removed and immediately frozen in liquid nitrogen and stored at −80°C for later biological or immunohistochemical analyses.

### 2.3. RNA Isolation and Gene Expression Analysis

Frozen tissue sections were minced in Trizol and further disrupted during 10 minutes of vigorous shaking. To extract the RNA, chlorophorm was added, mixed, and centrifuged. The aqueous phase containing the RNA was collected in a separate tube, and isopropanol was added. For precipitation, the RNA solution was centrifuged 15 minutes at 4°C at high speed. The RNA pellet was then further purified using the RNeasy Mini Kit (Qiagen) according to manufacturer's protocol. One *μ*g of RNA was reverse transcribed into cDNA using the High Capacity Kit (Applied Biosystems) and then further diluted to a final concentration of 5 ng/*μ*L cDNA.

The relative quantification of mRNA levels were carried out on a 7900 TaqMan systems (Applied Biosystem). To assess the mRNA expression of the target genes, real-time PCR was performed using 5 *μ*L of the gene expression master mix (Applied Biosystems) and 0.5 *μ*L of the gene expression assay for IL-1*β* (Mm00434228_m1), IL-6 (Mm00446190_m1), TNF-*α* (Mm00443258_m1), IL-10 (Mm00439616_m1), TGF-*β* (Mm00441724_m1), ANF (Mm01255747_g1), MMP13 (Mm00439491_m1), TIMP1 (Mm00441818_m1) (each includes forward and reverse primers as well the fluorescently FAM-labelled probe) from Applied Biosystems, and 1 *μ*L of cDNA in a final volume of 10 *μ*L. Quantification of the house keeping gene 18S (Hs99999901_s1) as an internal control was performed for each sample. Data were normalized to 18S RNA level as an endogenous control and are expressed using the formula 2^−ΔΔCt^ in comparison to the corresponding untreated controls. CVB3 copy numbers were detected using a forward primer (CCCTGAATGCGGCTAATCC) and a reverse primer (ATTGTCACCATAAGCAGCCA) in a final concentration of 60 ng/*μ*L as well as a FAM-labelled MGB probe (FAM-TGCAGCGGAACCG) in a final concentration of 5 pM.

### 2.4. Histological Measurements

Pieces of heart were either embedded in Tissue-Tek or paraffin. Sections embedded in Tissue-Tek were stained with antibodies directed against CD3 (goat anti-CD3; Santa-Cruz), VCAM (rat anti-VCAM; Pharmingen), collagen I (rabbit anti-ColI; Chemicon), and collagen III (rabbit anti-ColIII; Calbiochem). The paraffin sections were used for Mac3 staining using a specific antibody directed against Mac3 (rat anti-Mac3; Pharmingen).

For VCAM and Mac3 staining a biotinylated secondary rabbit anit-rat antibody and for CD3 staining a biotinylated secondary rabbit anti-goat antibody was used followed by visualization with a biotin-streptavidin-peroxidase technique (Vectorlabs). For visualization of ColI and ColIII staining, the Envision peroxidise technique was used (Dako).

### 2.5. In Situ Hybridization

Tissue sections from frozen hearts 28 days after CVB3 infection were used for detection of viral RNA with a ^35^S-labelled CVB3-specific RNA probe as described previously [17, 18]. Briefly, hybridisation with RNA probe proceeded at 42°C for 18 hours. Slices were then washed as described [18], and nonhybridized single-strand RNA probes were digested by RNase A. Slices were autoradiographed and stained with hematoxylin/eosin.

### 2.6. Statistical Analysis

Data are shown as mean ± SEM. For comparison the nonparametric, Mann-Whitney *U* test was used. Differences were considered significant when the probability value *P* is lower than 0.05. All analyses were performed using Graph Pad Prism 5.0 software (GraphPad Software, La Jolla, CA).

## 3. Results

### 3.1. Cytokine Expression after CVB3 Infection

To study the cytokine response induced by intraperitoneal CVB3 infection, the mRNA expression levels in cardiac tissue of infected and non infected WT and STAT3 KO mice were analysed.

**Figure 1:**
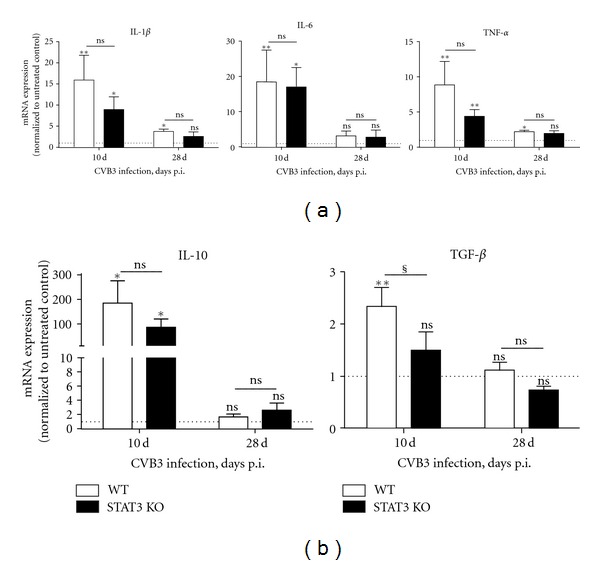
Cytokine expression levels in cardiac tissue of CVB3 infected mice. The mRNA expression levels are shown 10 and 28 days after CVB3 infection of WT and STAT3 KO mice. The expression data are normalized to the house-keeping gene 18S and to the expression levels of the corresponding untreated mice and expressed as x-fold over basal expression using the formula 2^−ΔΔCt^. *Data were compared to the expression of the corresponding untreated controls. *P < 0.05; **P < 0.01. ^§^Data between WT and STAT3 KO mice were compared. ^§^ *P* < 0.05.

10 days after infection with CVB3 WT mice show a significantly increased mRNA expression level of the proinflammatory cytokines IL-1*β* (15.92 ± 5.86 fold, *P* = 0.0043), IL-6 (18.62 ± 8.89 fold, *P* = 0.0043), and TNF-*α* (8.85 ± 3.34 fold, *P* = 0.0043) compared to the expression level in cardiac tissue of untreated WT mice. Whereas, 28 days after CVB3 infection WT mice show a weaker but still significantly increased mRNA expression level of IL-1*β* (3.75 ± 0.57, *P* = 0.0195) and TNF-*α* (2.20 ± 0.21, *P* = 0.0430) compared to the expression levels in untreated controls. A raised IL-6 mRNA expression level could no longer be detected in the CVB3-infected WT mice (Figure 1(a)—white bars). The anti-inflammatory cytokines IL-10 (184.41 ± 90.69 fold, *P* = 0.0357) and TGF-*β* (2.33 ± 0.37 fold, *P* = 0.0087) are both significantly increased 10 days after CVB3 infection, whereas no raised mRNA expression was determined 28 days after infection (Figure 1(b)—white bars).

In the cardiac tissue of infected STAT3 KO mice, the mRNA expression of the proinflammatory cytokines IL-1*β* (8.95 ± 3.00 fold, *P* = 0.0111), IL-6 (17.19 ± 5.39 fold, *P* = 0.0140), and TNF-*α* (4.39 ± 0.96, *P* = 0.0055) was significantly increased 10 days after infection compared to the expression levels in untreated STAT3 KO mice. In contrast to the cytokine expression in WT mice, the expression of IL-1*β* and TNF-*α* in STAT3 KO mice was not longer raised 28 days after infection. However, as already shown for infected WT mice, no increased IL-6 expression was determined (Figure 1(a)—black bars). The expression of the anti-inflammatory cytokine IL-10 (85.91 ± 33.96 fold, *P* = 0.0357) was increased 10 days after CVB3 infection and decreased to a normal expression level 28 days after infection. Interestingly, an augmented TGF-*β* expression was not detected in STAT3 KO mice 10 or 28 days after CVB3 infection (Figure 1(b)—black bars).

Comparing the cytokine expression of infected WT mice and infected STAT3 KO mice, only few differences were obvious. Interestingly, in infected STAT3 KO mice, TGF-*β* expression was not significantly increased (1.50 ± 0.35 fold, *P* = 0.2766) 10 days after infection, in contrast to the raised expression level in infected WT mice (2.33 ± 0.37 fold, *P* = 0.0087) (Figure 1(b)). Additionally, the anti-inflammatory cytokine IL-10, which is increased in both WT and STAT3 KO mice, is slightly but not significantly weaker increased in STAT3 KO mice 10 days after CVB3 infection.

### 3.2. Immune Cell Infiltration after CVB3 Infection

The intraperitoneal CVB3 infection leads to a significantly increased infiltration of CD3^+^ and Mac3^+^ cells in cardiac tissue of WT as well as STAT3 KO mice. In general, 10 days after CVB3 infection, more infiltrated cells were determined than 28 days after infection.

**Figure 2:**
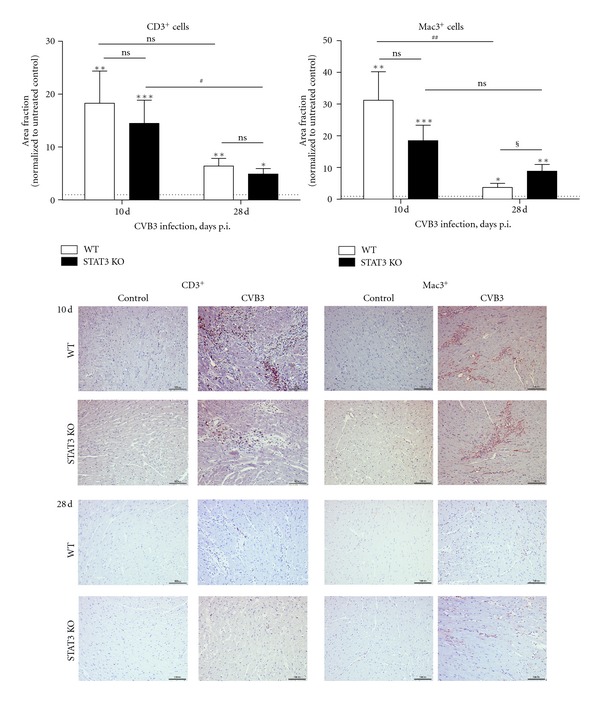
Cell infiltration in cardiac tissue 10 and 28 days after CVB3 infection. Data are expressed as area fraction of tissue sections after staining with antibodies directed against CD3 or Mac3. Data were normalized to corresponding untreated controls and expressed as x-fold over basal level. *Data were compared to the corresponding untreated controls. *P < 0.05; **P < 0.01; ***P < 0.001. ^§^Data between WT and STAT3 KO mice were compared. ^§^ *P* < 0.05. ^#^Data between 10 or 28 days were compared. ^#^ *P* < 0.05; ^##^ *P* < 0.01.

In WT mice, the number of infiltrated CD3^+^ (18.30 ± 6.06 fold, *P* = 0.0043) and Mac3^+^ (31.19 ± 9.02 fold, *P* = 0.0043) cells were significantly increased 10 days after CVB3 infection compared to the control animals. In comparison to the inflammation occurring 10 days after infection, the number of CD3^+^ cells was slightly but not significantly decreased to 6.40 ± 1.45 fold (*P* = 0.0070) compared to untreated controls 28 days after infection. In contrast, the number of infiltrated Mac3^+^ cells was strongly and significantly (*P* = 0.0046) reduced from 31.19 ± 9.02 fold 10 days after infection to 3.72 ± 1.29 fold (*P* = 0.028 compared to untreated controls) 28 days after infection (Figure 2—white bars).

In the cardiac tissue of infected STAT3 KO mice, a significantly increased number of CD3^+^ cells (14.48 ± 4.38 fold, *P* = 0.0003) was observed 10 days after CVB3 infection which was significantly reduced (*P* = 0.0379) to an 4.90 ± 1.03 fold increase (*P* = 0.0182) compared to untreated controls 28 days after infection. In contrast, the number of infiltrated Mac3^+^ cells 10 days after CVB3 infection (14.48 ± 4.86 fold, *P* = 0.0006) was slightly but not significantly reduced (8.86 ± 2.12 fold, *P* = 0.0091) 28 days after infection (Figure 2—black bars).

No differences were found comparing the number of infiltrated CD3^+^ cells between WT and STAT3 KO mice. Interestingly, the significantly reduced infiltration of Mac3^+^ cells in cardiac tissue of CVB3-infected WT comparing day 10 and 28 after infection could not be demonstrated in the cardiac tissue of infected STAT3 KO mice. There, the number of Mac3^+^ cells was not significantly decreased after 28 days compared to 10 days. Even more Mac3^+^ cells were found in cardiac tissue of CVB3-infected STAT3 KO mice compared to CVB3-infected WT mice (WT: 3.72 ± 1.29 fold versus STAT3 KO: 8.86 ± 2.12 fold, *P* = 0.0232) 28 days after infection.

### 3.3. Virus Load and Clearance

Intraperitoneal CVB3 infection resulted in a high viral genome quantity in cardiac tissue of both infected groups 10 days after infection determined by gene expression analysis. No viral genome was detected in non infected control animals. No significant difference was determined in viral genome quantity comparing infected WT mice ((151 ± 88) × 10^3^ copy numbers) and infected STAT3 KO mice ((30 ± 18) × 10^3^ copy numbers). Similarly, 28 days after infection, the copy number of viral genome in infected WT mice (18 ± 10) and infected STAT3 KO mice (16 ± 5) revealed no differences. Therefore, a nearly complete virus clearance was demonstrated for both WT and STAT3 KO mice 28 days after CVB3 infection. The complete virus clearance was further confirmed using *in situ* hybridisation which stains virus replication with a radioactively labelled CVB3-specific probe. There, no viral genome was detected in infected wild-type or STAT3 KO mice 28 days after infection.

### 3.4. Expression of Adhesion Molecule VCAM

**Figure 3:**
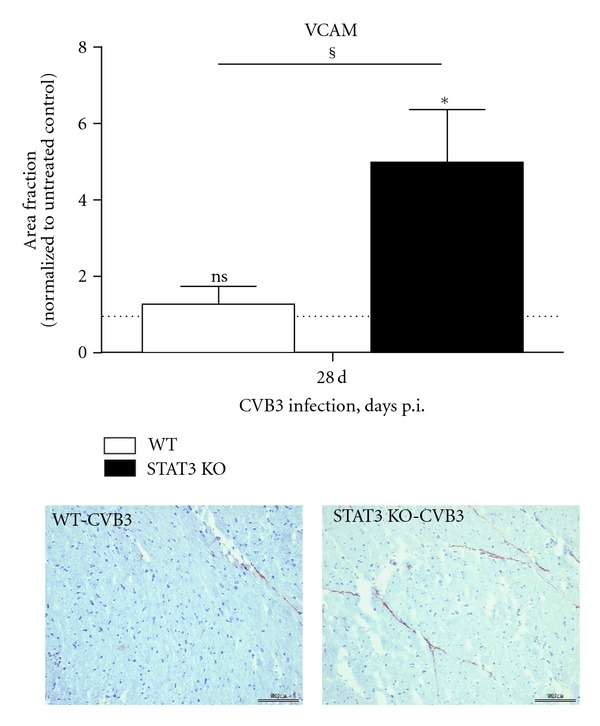
VCAM expression in cardiac tissue of CVB3 infected mice. The expression level was examined at cryosections of control or infected mice. The expression is shown as area fraction and was normalized to the corresponding untreated mice and expressed as x-fold over basal expression. *Data were compared to the expression of the corresponding untreated controls. *P < 0.05; ^§^Data between WT and STAT3 KO mice were compared. ^§^ *P* < 0.05.

The expression of the vascular cell adhesion molecule VCAM was analysed using cryosections of untreated and CVB3-infected WT and STAT3 KO mice 28 days after infection. Whereas the VCAM expression was not raised in infected WT mice compared to their untreated controls (1.28 ± 0.46 fold, *P* = 0.5536), a significant increase was determined in STAT3 KO mice (5.00 ± 1.38 fold, *P* = 0.0290). Therefore, the significantly higher VCAM expression in infected STAT3 KO mice compared to infected WT mice was obvious (*P* = 0.0250) (Figure 3).

### 3.5. Hemodynamic Data

The infected WT and the infected STAT3 KO mice were hemodynamically characterized 28 days after infection and compared to the hemodynamic function of their respective non infected controls.

As shown in Table 1, the global function in infected WT and infected STAT3 KO mice is restricted compared to the respective controls. Both WT and STAT3 KO revealed a significantly reduced cardiac output and stroke work induced by CVB3 infection. Furthermore, the ejection fraction is significantly reduced in infected STAT3 KO mice and slightly but not significantly reduced in infected WT mice. Moreover, CVB3 infection resulted in impaired systolic and diastolic function indicated by significantly reduced P_es_ and dP/dt_max⁡_ as well as significantly increased P_ed_, dP/dt_min⁡_, and Tau in WT as well as in STAT3 KO mice. 

Interestingly, compared to their respective controls, infected STAT3 KO mice reveal a significantly more impaired global, systolic, and diastolic function. The ejection fraction is reduced to 78% in infected WT animals and significantly more decreased to 57% in infected STAT3 KO mice. Moreover, the end systolic pressure P_es_ was reduced to 87% in infected WT and to 60% in infected STAT3 animals. Furthermore, the end diastolic pressure P_ed_ was 2.7-fold higher in infected WT mice and even 5-fold increased in infected STAT3 KO mice compared to their respective controls.

### 3.6. ANF as Marker for Heart Failure

**Figure 4:**
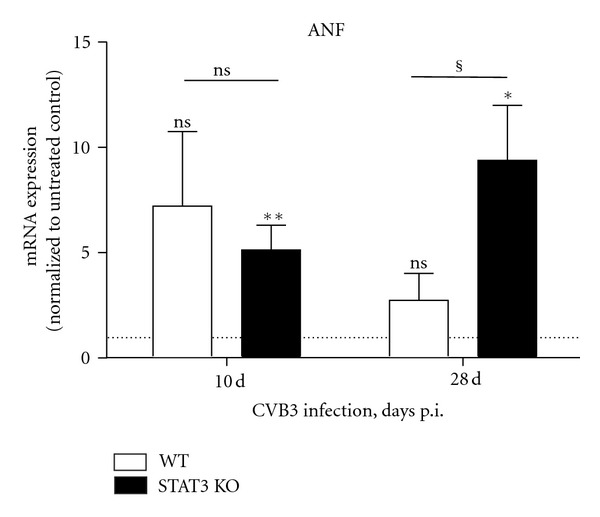
ANF expression in cardiac tissue of CVB3 infected mice. The mRNA expression level is shown 10 and 28 days after CVB3 infection of WT and STAT3 KO mice. The expression data are normalized to the house-keeping gene 18S and to the expression levels of the corresponding untreated mice and expressed as x-fold over basal expression using the formula 2^−ΔΔCt^. *Data were compared to the expression of the corresponding untreated controls. *P < 0.05; **P < 0.01. ^§^Data between WT and STAT3 KO mice were compared. ^§^ *P* < 0.05.

Since atrial natriuretic factor (ANF) is known as a marker for heart failure, we examined the mRNA expression level of ANF which was higher expressed in cardiac tissue of infected WT (7.20 ± 3.54 fold, *P* = 0.0823) and STAT3 KO (5.11 ± 1.18 fold, *P* = 0.0079) mice as in control animals. Interestingly, in infected WT mice, ANF expression was nearly reduced to the normal expression level (2.72 ± 1.29 fold, *P* = 0.9371) 28 days after infection. Whereas, the increased ANF expression level remained unchanged in STAT3 KO mice (9.37 ± 2.62 fold, *P* = 0.0476) and revealed a significant higher expression (*P* = 0.0160) of ANF in cardiac tissue of infected STAT3 KO mice compared to infected WT 28 days after infection (Figure 4).

### 3.7. Extracellular Matrix Alteration

Regarding the regulation of extracellular matrix cryosections of cardiac tissue from CVB3 infected and not infected mice were stained with antibodies directed against collagen I or collagen III.

**Figure 5:**
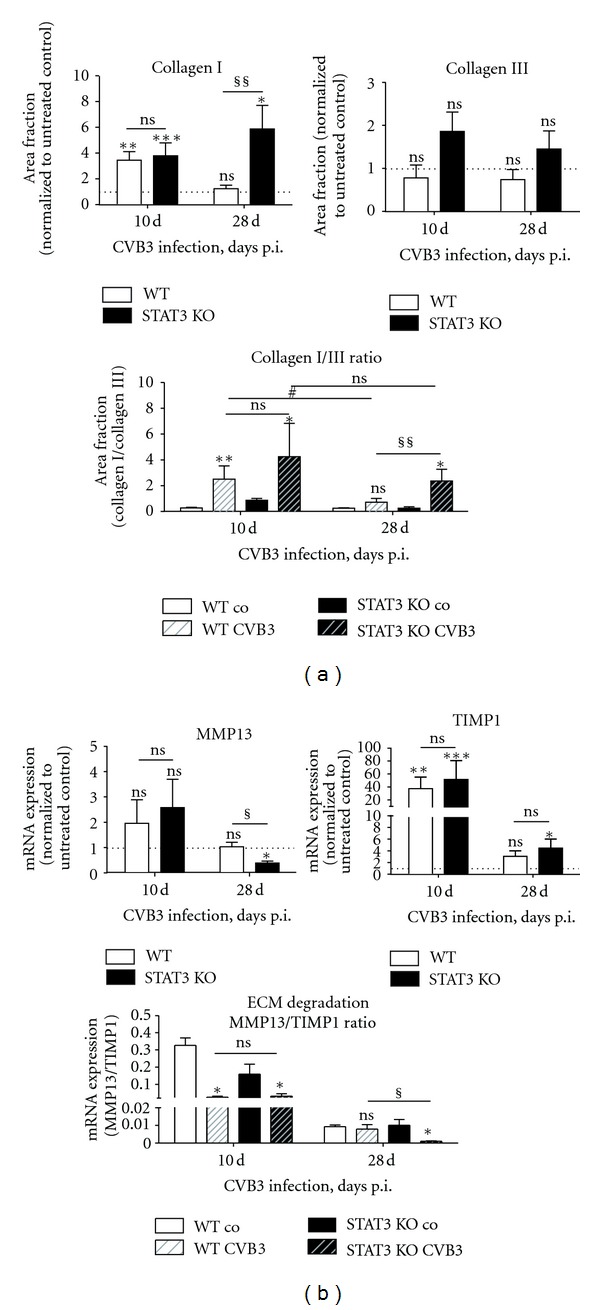
Collagen expression and expression of the ECM degrading system in cardiac tissue of CVB3-infected mice. The area fractions of collagen I and collagen III are shown 10 and 28 days after CVB3 infection of WT and STAT3 KO mice as x-fold compared to the corresponding untreated controls. The mRNA expression data of MMP13 and TIMP1 are normalized to the house-keeping gene 18S and to the expression levels of the corresponding untreated mice and expressed as x-fold over basal expression using the formula 2^−ΔΔCt^. The ratios were calculated without previous normalization to the untreated controls. * Data were compared to the expression of the corresponding untreated controls. *P < 0.05; **P < 0.01; ***P < 0.001. ^§^Data between WT and STAT3 KO mice were compared. ^§^ *P* < 0.05; ^§§^ *P* < 0.01. ^#^Data between 10 or 28 days were compared. ^#^ *P* < 0.05.

No increased area fraction of collagen III was determined 10 or 28 days after infection, whereas collagen I deposition in the cardiac tissue was induced by CVB3. 10 days after infection, significantly increased collagen I content was measured in both infected WT (3.45 ± 0.66 fold, *P* = 0.0043) and infected STAT3 KO mice (3.79 ± 1.00 fold, *P* = 0.0006). Interestingly, 28 days after infection, the collagen I content in infected WT mice (1.24 ± 0.27 fold) was reduced to the collagen I level comparably to non infected control animals. Whereas, this reduced collagen I content could not be detected in infected STAT3 KO mice 28 days after infection. There, the area fraction of collagen I was still significantly increased by 5.88 ± 1.82 fold compared to the untreated controls which revealed a significant difference between infected WT and infected STAT3 KO mice (*P* = 0.0014). Furthermore, the Col I/ColIII ratio displays a CVB3-induced increase 10 days after infection. In WT mice, this increase dropped significantly down to the normal level 28 days after infection, whereas, in infected STAT3 KO mice, the CVB3-induced increase remains unchanged 28 days after infection which reveals a significant distinction (*P* = 0.0077) between infected WT and infected STAT3 KO mice (Figure 5(a)). Consequently, CVB3 infection resulted in increased fibrosis in STAT3 KO compared to WT mice.

Additionally, we further examined the mRNA expression levels of the ECM-degrading system. The mRNA expression of the collagenase MMP13 was not significantly increased 10 days after infection, whereas the expression of the endogenous inhibitor TIMP1 was significantly increased (WT: 37.42 ± 17.78 fold, *P* = 0.0043; STAT3 KO: 51.59 ± 28.97 fold, *P* < 0.0001) which is then reduced to an only slightly increased expression 28 days after infection (WT: 3.07 ± 0.93 fold, *P* = 0.2168; STAT3 KO: 4.47 ± 1.54 fold, *P* = 0.0485) and revealed no distinction between WT and STAT3 KO mice. In contrast, the mRNA expression of MMP13 in STAT3 KO mice is significantly reduced (0.39 ± 0.08 fold, *P* = 0.0121) 28 days after CVB3 infection, whereas the MMP13 expression in infected WT mice remains unchanged.

Concerning the MMP13/TIMP1 ratio, the CVB3-induced significant reduction of ECM degradation is clearly demonstrated for WT and STAT3 KO mice 10 days after infection but revealed no difference between both. Interestingly, this inhibition of ECM degradation was still demonstrated in infected STAT3 KO mice 28 days after infection but was not longer determined in infected WT animals.

## 4. Discussion

To study the relevance of the signal transducer and activator of transcription molecule 3 (STAT3) in CVB3-induced myocarditis, we examined mice with a cardiomyocyte-restricted STAT3 deletion. We show for the first time that STAT3 KO induces adverse cardiac remodelling leading to DCM in the subacute phase of viral myocarditis, while no change was seen in the acute phase when cardiomyocyte-restricted STAT3 KO was compared to wild-type. This was interestingly associated with increased cardiac inflammation followed by an exaggerated remodelling process in cardiomyocyte-restricted STAT3 KO mice and deregulating the matrix degradation system.

Acute CVB3 infection leads to a robust inflammation in cardiac tissue of wildtype mice, which is demonstrated by high numbers of infiltrated inflammatory cells and highly increased expression of proinflammatory cytokines such as IL-1*β*, IL-6, and TNF-*α* 10 days after infection [1, 19, 20]. It is previously described for the mouse strain C57/BL6j that the virus does not induce a chronic ongoing inflammation [20] and animals recover from myocarditis. In line with these findings, the wild-type animals show a reduced number of invaded cells and decreased mRNA expression of cytokines 28 days after viral infection. Furthermore, nearly a complete virus clearance was detected 28 days after infection. Controlling inflammation therefore was associated with no adverse cardiac remodelling which can be demonstrated by no collagen accumulation as well as nearly normal LV function 28 day after infection in wild-type animals.

STAT3 is well known as a transcription activator of IL-6 [21, 22]. Since the STAT3 KO is restricted to cardiomyocytes [14], in cardiac tissue of infected STAT3 KO mice, a highly upregulated IL-6 expression was detected due to the infiltration of inflammatory cells still expressing STAT3 in this animal model. Compared to the infected wild-type mice, the mRNA expression levels of IL-1*β*, IL-6, and TNF-*α* as well as the number of infiltrating immune cells revealed no distinction in STAT3 KO mice 10 days after infection. While inflammation was controlled and therefore resolved in wild-type animals between day 10 and 28, in STAT3 KO mice, the number of invaded Mac3^+^ cells was not reduced significantly after the acute phase despite viral genome was also extinguished. The finding that endothelial activation demonstrated as increased vascular cell adhesion molecule (VCAM) expression level on endothelial cells in cardiac tissue of STAT3 KO mice is increased accommodates with the unchanged number of infiltrated Mac3^+^ cells found 28 days after infection. The specific effects of cardiomyocyte-restricted STAT3 KO on endothelial VCAM expression levels have to be revealed in future studies, but it is intriguing to speculate that altered myocyte to endothelial crosstalk may be involved in this upregulation of VCAM and therefore fuel cardiac inflammation.

The previously reported characterisation of the cardiomyocyte-restricted STAT3 KO mice in comparison to wild-type mice revealed development of cardiac fibrosis in aging KO mice which was associated with the impaired cardiac function [14, 15]. Left ventricles of aging wild-types and STAT3 KO reveal an increased expression of profibrotic genes such as collagen-I*α*1, connective tissue growth factor (CTGF), and TIMP1 which could be the reason for the age-dependent interstitial fibrosis [14]. Here, 10 days after CVB3 infection the wild-type and STAT3 KO animals revealed a similar increase of interstitial collagen I in cardiac tissue, whereas the amount of collagen III was not affected by CVB3 resulting in an increased Col I/Col III ratio. This reveals that cardiac inflammation which controls cardiac remodelling was not differently regulated in the acute phase of myocarditis in this animal model.

However, this increased Col I/Col III ratio declined to normal levels in infected wild-type mice 28 days after infection. In contrast, in STAT3 KO mice the CVB3 infection resulted in a Col I/Col III ratio still being upregulated after 28 days. This ongoing fibrosis in infected STAT3 KO resulted in impaired cardiac function, since collagen is known to depress cardiac function in experimental models as well as in patients with cardiomyopathies [23, 24].

To further investigate the distinct mechanisms of this changed remodelling in response to inflammation, we investigated the regulation of the matrix degradation system [25].

In the acute phase, an increased expression of TIMP1, which is an endogenous inhibitor of the ECM-degrading matrix metalloproteinases [26, 27], prevents collagen degradation and thus revealed matrix deposition in both wild-type and STAT3 KO mice 10 days after infection. One of the most abundant matrix metalloproteases in the cardiac tissue is the collagenase MMP13. Interestingly, reduced MMP13 expression found in infected STAT3 KO mice 28 days after infection is consistent with the increased collagen I protein content in infected STAT3 KO mice 28 days after infection. The deposition and degradation of ECM is a finely balanced equilibrium between the degrading enzymes and their endogenous inhibitors [28, 29]. To clarify the ECM degradation activity in the infected cardiac tissue, the MMP13/TIMP1 ratio reveals a significantly reduced degradation activity in infected STAT3 KO mice 28 days after infection compared to the infected wild-type animals. Concerning the influence of the cardiomyocyte-restricted STAT3 KO on the regulation of ECM, it could be assumed that cardiomyocytes release paracrine factors to influence the ECM regulation. The presence of those paracrine factors was confirmed by the finding that the cell culture supernatant of isolated cardiomyocytes from STAT3 KO animals induced a higher fibroblast proliferation compared with wild-type cardiomyocytes supernatant, as shown earlier [14]. Since cardiac fibroblasts are the most prominent producers of ECM proteins as well as of the ECM degradation system, the paracrine effects shown for fibroblast proliferation can also be assumed for regulation of ECM deposition or degradation by cardiac fibroblasts [30–33].

Conventional knockout of the STAT3 gene leads to embryonic lethality at embryonic day 6.5 [13]. Therefore, the cardiomyocyte-restricted KO was chosen to study the protective function of STAT3 against CVB3-induced myocarditis *in vivo*. It has already been shown that the IL-6 cytokine family using the Jak/STAT pathway protects cardiomyocytes from apoptotic cell death in response to serum starvation or ischemia and induces hypertrophy in cardiomyocytes [34, 35]. In previous studies, the cardiac function of the cardiomyocyte-restricted STAT3 KO mice has been analysed. At a young age, the cardiac structure and function are apparently normal but an age-related increase in cardiac apoptosis and fibrosis has been described [14, 15]. The cardiomyocyte-restricted deletion of receptor subunit gp130, which also prevents STAT3 signalling, also leads to a dilated ventricle after pressure overload [36].

In the present study, we used CVB3 to induce heart failure. The hemodynamic characterisation clearly shows a significantly reduced cardiac function of CVB3-infected STAT3 KO mice compared to CVB3-infected wild-type mice 28 days after infection. These findings are in line with the described cardiac dysfunction of cardiomyocyte-restricted STAT3 KO mice after myocardial infarction or doxorubicin-induced cardiomyopathy [14, 15]. After myocardial infarction, the KO mice revealed a larger infarct size as well as a more pronounced deterioration in systolic dysfunction [14]. In another study, they demonstrated that animals with the cardiomyocyte-restricted STAT3 KO are more susceptible to doxorubicin-induced cardiac injury and develop heart failure. Thus, STAT3 deletion leads to impaired cardiac function after myocardial infarction and doxorubicin-induced cardiomyopathy. Here, we demonstrate for the first time that STAT3 deletion also leads to an aggravated cardiac function in viral myocarditis induced by CVB3. Furthermore, the cardiac-specific overexpression of STAT3 in transgenic mice protected against doxorubicin-induced apoptosis and therefore is another evidence that STAT3 may protect hearts from injuries caused by different stressors [37].

In conclusion, the present study revealed new insights in the protective function of STAT3 expressed in cardiomyocytes after CVB3-induced myocarditis. There and in other cardiac damages such as myocardial infarction or doxorubicin-induced cardiomyopathy, STAT3 in cardiomyocytes prevents uncontrolled fibrosis and clinical progression to DCM. Therefore, STAT3 seems to be a crucial factor for the resolution of viral myocarditis.

## Figures and Tables

**Table 1:** Animal characteristics and hemodynamic measurements 28 days after CVB3 infection.

	WT	WT-CVB3	STAT3 KO	STAT3 KO-CVB3
Body weight [g]	29 ± 2	23 ± 1	28 ± 1	22 ± 1
Heart weight [g]	0.15 ± 0.03	0.09 ± 0.01	0.11 ± 0.01	0.09 ± 0.01
Global function				
Heart rate [bpm]	434 ± 30	404 ± 14	353 ± 90	420 ± 12
Cardiac output [mL/min]	15 ± 1	11 ± 1*	14 ± 1	8 ± 1^∗†^
Stroke volume [*μ*L]	31 ± 4	29 ± 2	30 ± 1	22 ± 2
Stroke work [*μ*L · mmHg]	3589 ± 116	2384 ± 117**	3331 ± 173	1631 ± 125^∗††^
Ejection fraction [%]	67 ± 3	52 ± 2	68 ± 2	39 ± 2^∗∗††^
Systolic function				
P_es_ [mmHg]	123 ± 6	100 ± 1*	122 ± 11	74 ± 9^∗†^
dP/dt_max⁡_ [mmHg/s]	9640 ± 786	6663 ± 325*	9415 ± 637	3422 ± 202^∗∗††^
V_es_ [*μ*L]	28 ± 2	31 ± 3	26 ± 2	47 ± 5^∗†^
Diastolic function				
P_ed_ [mmHg]	4.0 ± 0.8	11.0 ± 1.2*	3.7 ± 0.3	18.7 ± 3.0^∗†^
dP/dt_min⁡_ [mmHg/s]	−7873 ± 319	−4757 ± 240**	−7813 ± 131	−2818 ± 216^∗∗††^
Tau [ms]	11.7 ± 0.3	15.2 ± 0.4*	11.3 ± 0.7	17.8 ± 0.8^∗∗†^
V_ed_ [*μ*L]	49 ± 5	57 ± 3	48 ± 3	74 ± 4^∗∗††^

*Significantly different versus respective control.

^†^Significantly different versus WT-CVB3.
